# Calprotectin as a diagnostic marker for sepsis: A meta-analysis

**DOI:** 10.3389/fcimb.2022.1045636

**Published:** 2022-11-28

**Authors:** Rong-Yue Gao, Hui-Miao Jia, Yu-Zhen Han, Ben-Shu Qian, Pan You, Xiao-Ke Zhang, Wen-Xiong Li, Li-Feng Huang

**Affiliations:** Department of Surgical Intensive Care Unit, Beijing Chao-yang Hospital, Capital Medical University, Beijing, China

**Keywords:** calprotectin, sepsis, diagnosis, meta-analysis, biomarker

## Abstract

**Introduction:**

Sepsis is a life-threatening condition, and biomarkers are needed to diagnose sepsis fast and accurately. We aimed to perform this meta-analysis to investigate the diagnostic value of calprotectin on sepsis in critically ill patients.

**Methods:**

The investigators searched MEDLINE, Embase, Web of Science and Cochrane Library. Studies were included if they assessed the diagnostic accuracy of serum calprotectin for sepsis in intensive care unit (ICU). We estimated its diagnostic value and explored the source of heterogeneity. The bivariate model and the hierarchical summary receiver operating characteristic (HSROC) curve were used in the meta-analysis.

**Results:**

Six records assessing 821 patients were included in this meta-analysis. The pooled sensitivity, specificity, positive likelihood ratio (PLR), and diagnostic odds ratio (DOR) were separately as 0.77, 0.85, 5.20, 0.27, respectively. The Fagan's nomogram showed post‐test probabilities of 91% and 35% for positive and negative outcomes, respectively. Subgroup analysis indicated that sepsis definition could be a possible source of heterogeneity, but there’s no sufficient data to investigate sepsis-3 definition. Sensitivity analysis suggested that two studies could affect the stability of pooled results.

**Conclusion:**

On the basis of our meta-analysis, calprotectin is a helpful marker for early diagnosis of sepsis on ICU admission.

## Introduction

Sepsis is a leading cause of mortality and critical illness worldwide and is defined as a life-threatening organ dysfunction caused by a dysregulated host response to infection (Vincent et al., 2014; Singer et al., 2016; Fleischmann et al., 2016). The Sequential Organ Failure Assessment (SOFA) score helps to identify patients with sepsis and is an important part of the current sepsis definition. However, it is still difficult to prove infection fast and accurately at complicated medical situations, and diagnosis of sepsis is often delayed (Taylor et al., 2021). Delay in the diagnosis and treatment of sepsis increases mortality and the financial burden (Bui et al., 2022; Souza et al., 2022). Moreover, the lack of a generally accepted biomarker golden standard for sepsis is a common limitation in all sepsis studies. Including a uniform group of patients is a challenge, thus restricting the practicality and catholicity of the results.

Therefore, biomarkers may play a vital role in the timely diagnosis and management of sepsis. C-reactive protein (CRP) and procalcitonin (PCT) are widely used biomarkers, but their diagnostic accuracy has been challenged (Wu et al., 2017; Wu et al., 2018; Yeh et al., 2019). It is reported that surgery and trauma can interfere with the results even in patients without infection (Parli et al., 2018). They cannot readily distinguish infection from sterile inflammation. Therefore, biomarkers with high diagnostic sensitivity and specificity are indispensable.

Calprotectin is a calcium-binding protein that was originally found in neutrophils and was named major leukocyte protein L1. It is a 24-kDa heterodimer composed of light [myeloid-related protein 8 (MRP8)] and heavy (MRP14) chains (8 and 14 kDa), also named S100A8 and S100A9, which belong to the S100 protein family (Edgeworth et al., 1991). Recent findings showed that calprotectin is closely related to sepsis. Increased ascitic calprotectin is associated with spontaneous bacterial peritonitis, and its most common etiology of mortality is sepsis (Abdel-Razik et al., 2020). Moreover, calprotectin in the amniotic fluid is a strong predictor of neonatal sepsis (Delanghe and Speeckaert, 2015). It was also reported of diagnostic value in sepsis-associated encephalopathy and sepsis-induced acute kidney injury (Zhang et al., 2016; Liao et al., 2021). The serum calprotectin expression level was also found significantly increased in sepsis and had a high diagnostic value (Canani et al., 2011; Gao et al., 2015; Decembrino et al., 2015; Huang et al., 2016; Simm et al., 2016; Shams et al., 2017; Wirtz et al., 2020; Larsson et al., 2020; Pirr et al., 2021). However, the number of patients in each study is limited and most of the studies recruited patients from a single center, leading to a potential lack of generalizability of the results. Moreover, the results vary in previous studies (sepsis vs. non-sepsis controls: 42.5%–89% sensitivity, 56%–96% specificity).

In consequence, we aimed to perform this meta-analysis to synthesize the current evidence and investigate the diagnostic value of calprotectin on sepsis.

## Materials and methods

### Data sources and search strategy

We constructed a protocol of complete meta-analysis that adhered to the Preferred Reporting Items for Systematic Reviews and Meta-Analyses (PRISMA) standards (Page et al., 2021). The protocol was registered with the PROSPERO database (registration number CRD42022304473).

The investigators searched MEDLINE, Embase, Web of Science, and Cochrane Library using the search term “(sepsis OR “bacterial infection” OR “systemic inflammatory response syndrome”) AND (calprotectin).” Two investigators (R-YG and Y-ZH) independently searched the databases from the establishment of these databases until January 2022 and reran the search before the final analysis. The research was done without language or publication period restrictions, and unpublished studies were not searched.

### Study selection criteria

Studies that assessed the accuracy of serum calprotectin for differentiation between critically ill patients with sepsis and those without sepsis were included. Sepsis could be diagnosed using any recognized diagnostic criteria, and there are no restrictions on the types of study design eligible for inclusion. All retrieved articles were initially screened by title and abstract. Subsequently, the relevant ones were rescreened by full text. The selection was done independently by two investigators (R-YG and Y-ZH). If there were any discrepancies in the process of the study selection, they would turn to a third party. EndNote was used for recording decisions.

### Data extraction

Two investigators (R-YG and Y-ZH) independently extracted the data from individual reports, including first author, year of publication, study location, population setting, admission category (surgical or medical), sepsis definition, and sample size. We also recorded true positive (TP), false positive (FP), false negative (FN), true negative (FN), sensitivity, specificity, area under the curve (AUC), and optimal cutoff value of calprotectin for the diagnosis of sepsis. If there was any disagreement between the two reviewers in the process of data extraction, it was resolved by a third party. We contacted the corresponding authors when further information was needed. If no response was received after sending a reminder, the study would be excluded. The data extracted were recorded in an Excel spreadsheet.

### Quality assessment

The quality assessment was done by two independent investigators using the Quality Assessment of Diagnostic Accuracy Studies 2 (QUADAS-2) tool (Whiting et al., 2011). This evaluation includes the following domains: patient selection, index test, reference standard, and flow and timing.

### Statistical analysis

Hierarchical models are increasingly being accepted as standard methods for the meta-analysis of diagnostic test accuracy studies (Lee et al., 2015). Therefore, the hierarchical summary receiver operating characteristic (HSROC) curve was computed, and the bivariate random effects model was used to determine the summary estimates of the sensitivity, specificity, diagnostic odds ratio (DOR), positive likelihood ratio (PLR), and negative likelihood ratio (NLR). Fagan’s nomogram was plotted to assess the utility of serum calprotectin for the diagnosis of sepsis.

Heterogeneity induced by threshold effect was calculated by testing Spearman correlation, and *p* < 0.05 represented significant threshold effects. The *I²* statistics was used to evaluate heterogeneity induced by a non-threshold effect. Different *I²* values reflected low (<30%), moderate (30%–50%), and high (>50%) degrees of heterogeneity.

Subgroup analyses were performed according to varied factors to identify the causes of heterogeneity. To examine the stability of the results, sensitivity analysis was also performed. It was analyzed with a method of reducing one article at a time, and the effect of a single study on the meta-analysis was evaluated. Publication bias was examined by Deeks’ funnel plot asymmetry test. All statistical analyses were performed using MetaDiSc (version 1.4) and STATA (version 15.1) software.

## Results

### Study selection and characteristics

**Figure 1:**
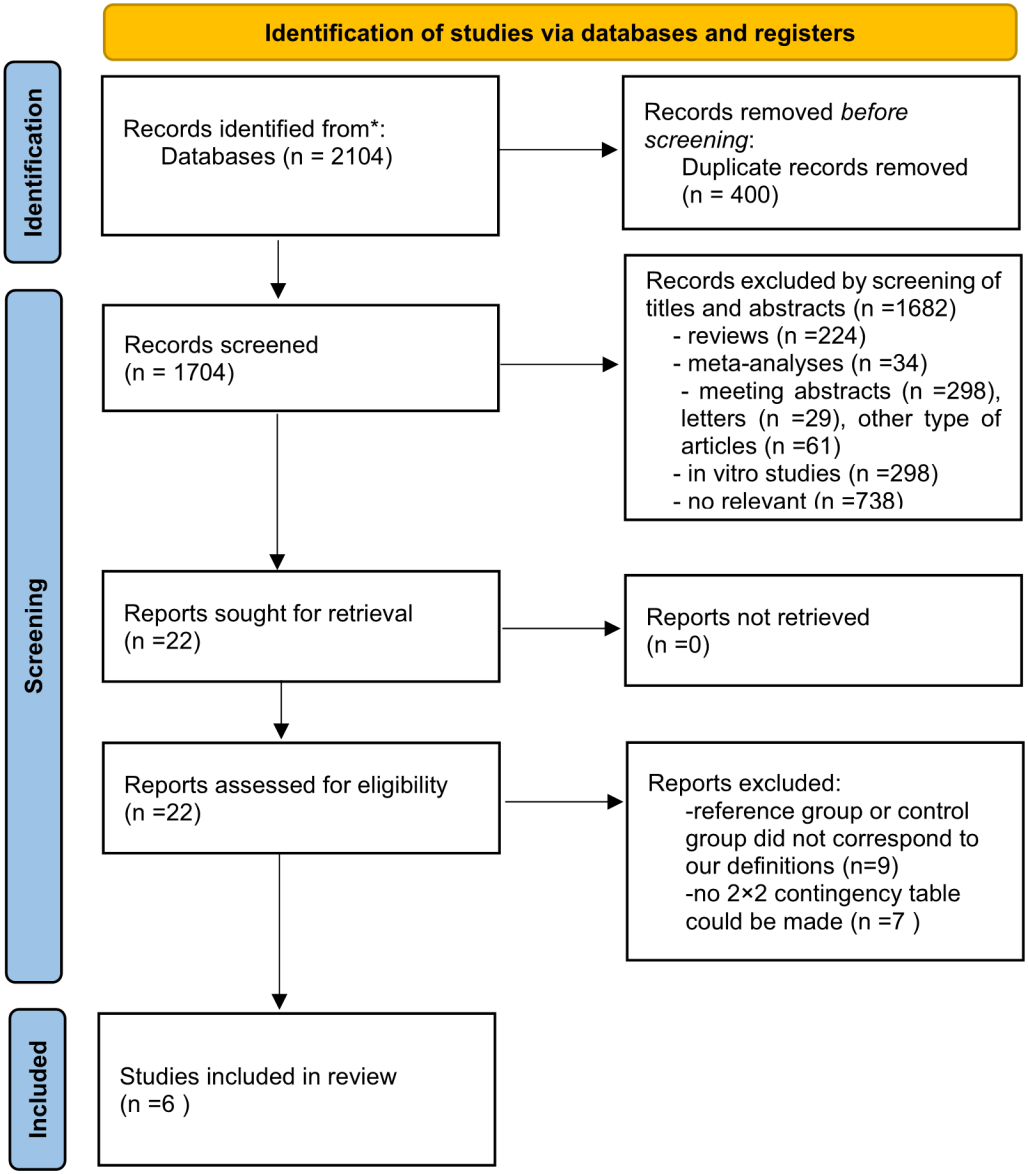
Preferred Reporting Items for Systematic Reviews and Meta-Analyses (PRISMA) flow diagram of the study selection.

A PRISMA flow diagram summarizing the study selection process is presented in **Figure 1**. A total of 2,104 related reports were initially obtained from the databases. After removing 2,082 animal or *in vitro* studies, duplicates, and irrelevant articles, a total of 22 full-text references were screened for eligibility. Among them, nine did not meet our selection criteria, and seven had insufficient data to construct a 2 × 2 contingency table. Finally, six records were included in our meta-analysis (Canani et al., 2011; Decembrino et al., 2015; Gao et al., 2015; Simm et al., 2016; Shams et al., 2017; Larsson et al., 2020). In two studies (Simm et al., 2016; Larsson et al., 2020), investigators reported the diagnostic accuracy separately for medical and surgical patients. The studies were divided into parts, but there were overlaps between different parts. Thus, we chose the datasets showing the highest Youden index for analysis. While in the subgroup analyses, we analyzed medical and surgical subgroups according to the admission category.

The characteristics of these studies are listed in **Table 1**. All eligible studies were published between 2011 and 2020, and most were done in Europe. All of these studies were conducted in intensive care units (ICUs), and blood samples were taken on admission to the ICU. Three of them investigated adult patients, whereas the other three were done in neonatal ICUs. As sepsis criteria changed in these years, two studies used the American College of Chest Physicians/Society of Critical Care Medicine (ACCP/SCCM) definition for sepsis, one study used the sepsis-3 definition, and the neonatal studies diagnosed sepsis by either clinical signs or blood culture. The cutoff for calprotectin concentration varied, ranging from 1,300 to 38,300 ng/ml.

**Table 1:** Summary of the included studies.

Author	Year	Country	Population	Admission Category	Sepsis Definition	Sample	Cutoff (ng/ml)	Sample Size	AUC	TP	FP	FN	TN	Sensitivity %	Specificity %
Canani	2011	Italy	Neonatal	Medical	Blood culture/Clinical	Serum	1,700	91	NR	55	1	7	28	89.0	96.0
Decembrino	2015	Italy	Neonatal	Medical	Blood culture	Serum	2,200	41	0.61	5	10	3	23	62.5	69.7
Gao	2015	China	Adult	NR	ACCP/SCCM	Plasma	3,128.8	298	0.9	188	8	38	64	83.1	88.5
Larsson	2020	Sweden	Adult	Trauma	Sepsis-3	Plasma	1,300	109	0.79	62	10	15	22	81.0	70.0
Larsson	2020	Sweden	Adult	Medical	Sepsis-3	Plasma	1,300	159	0.67	62	34	15	48	81.0	59.0
Shams	2017	Iran	Neonatal	Medical	Clinical	Plasma	38,300	80	NR	17	4	23	36	42.5	90.0
Simm	2016	Sweden	Adult	Surgical	ACCP/SCCM	Plasma	3,400	38	0.65	9	10	6	13	60.0	56.5
Simm	2016	Sweden	Adult	Medical	ACCP/SCCM	Plasma	2,700	22	0.95	13	1	2	6	86.7	85.7

AUC, area under the curve; TP, true positive; FP, false positive; FN, false negative; TN, true negative; NR, not reported; ACCP/SCCM, American College of Chest Physicians/Society of Critical Care Medicine; ELISA, enzyme-linked immunosorbent assay; PETIA, particle-enhanced turbidimetric immunoassay.

### Quality assessment and publication bias

**Figure 2:**
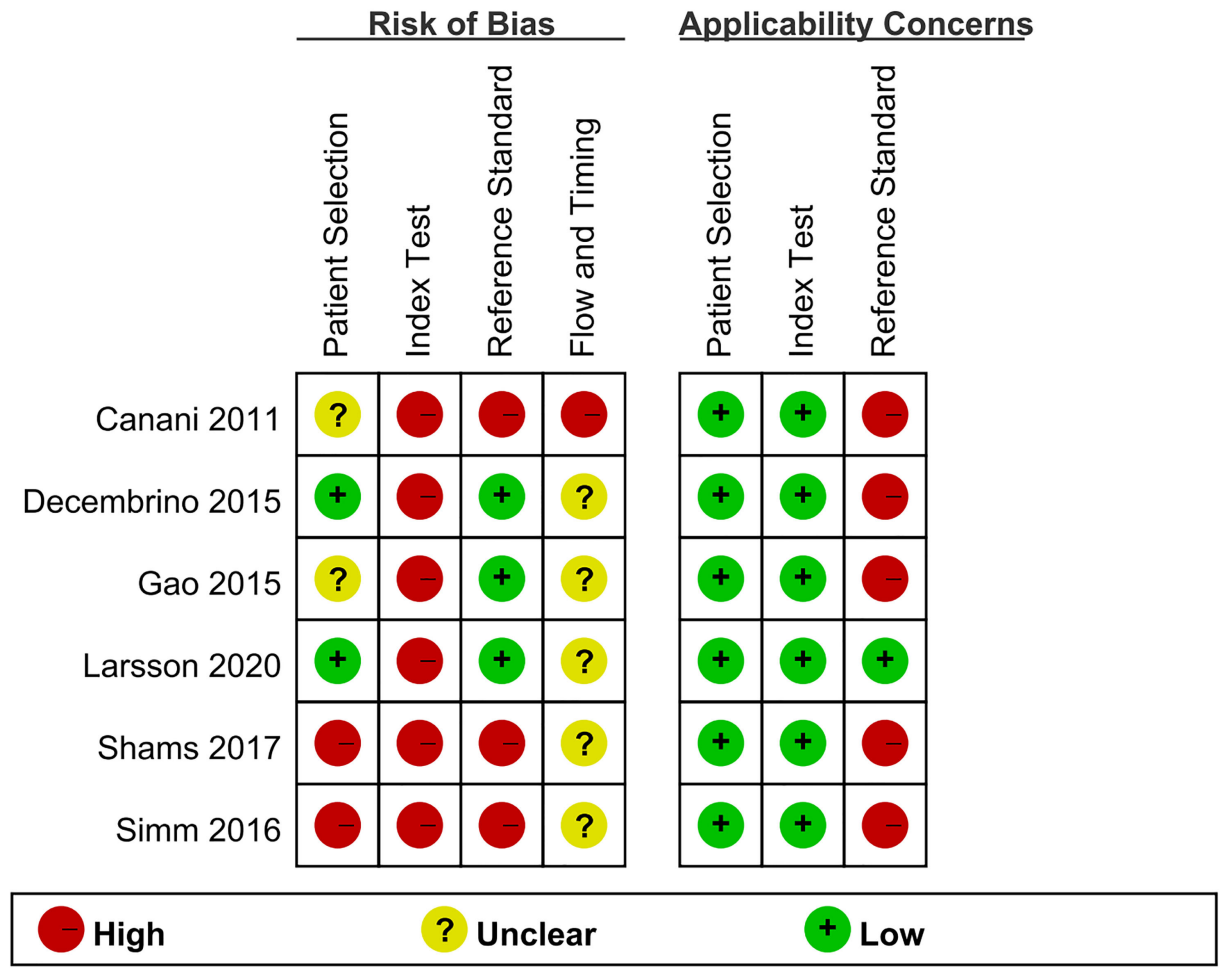
Risk of bias and applicability concerns of the included studies.

**Figure 3:**
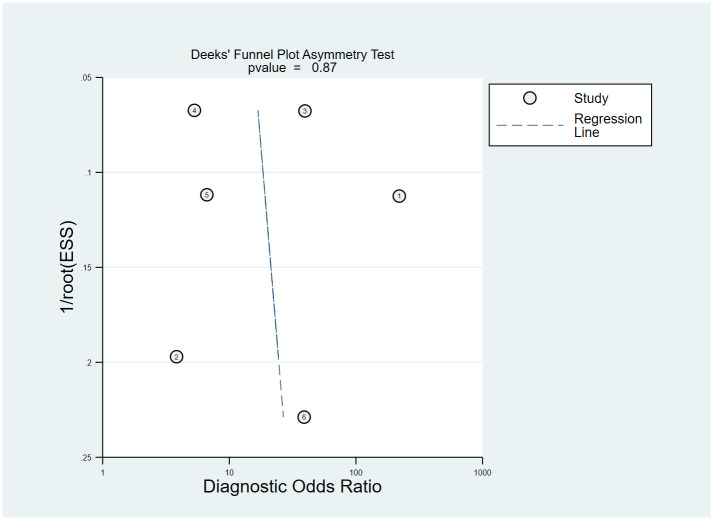
Deeks’ funnel plot.

The QUADAS-2 tool was applied to evaluate the quality of the six studies, and the detailed results were shown in **Figure 2**. Deeks’ funnel plot asymmetry test (**Figure 3**) results indicated that no significant publication bias existed in this meta-analysis (*p* = 0.87).

### Data synthesis

**Figure 4:**
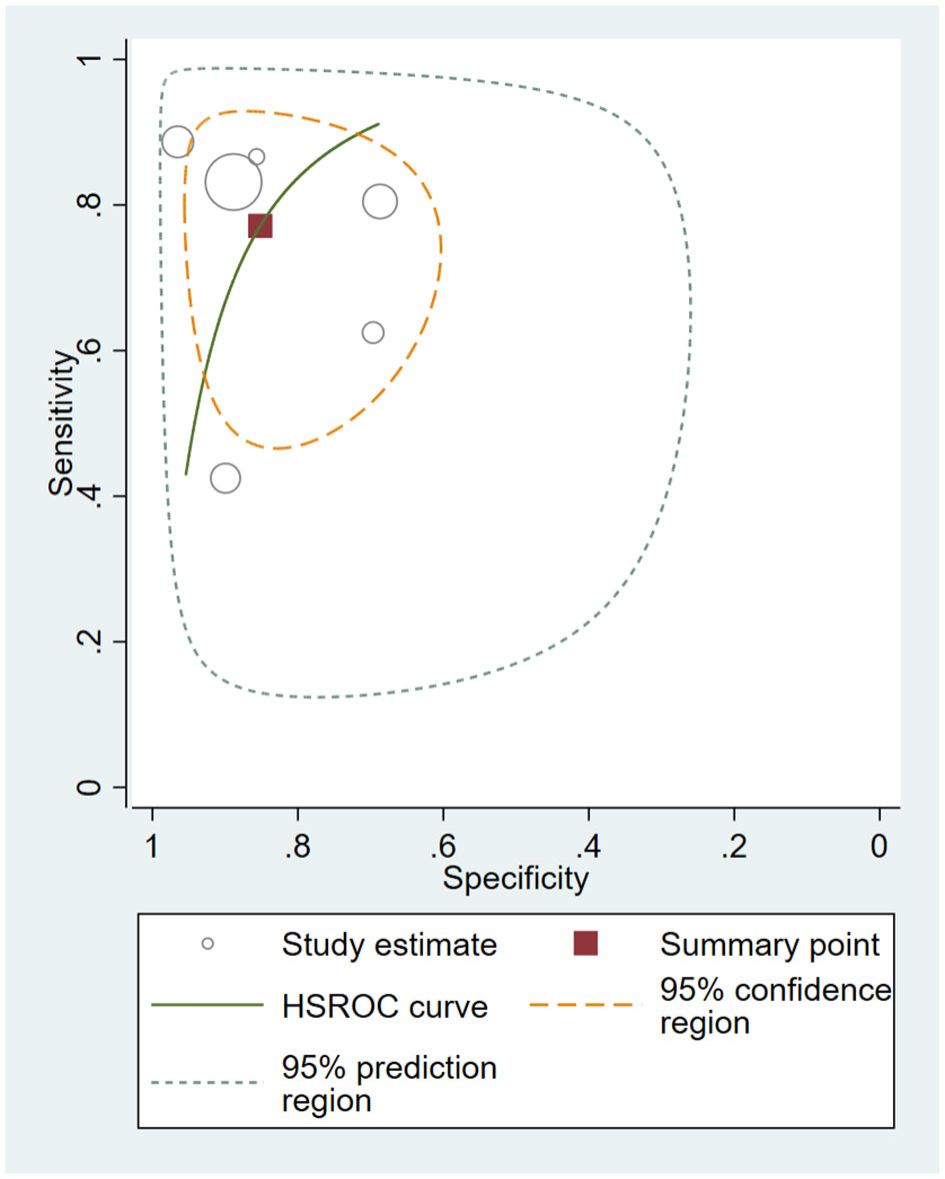
Hierarchical summary receiver operating characteristic (HSROC) curves for all included studies.

**Figure 5:**
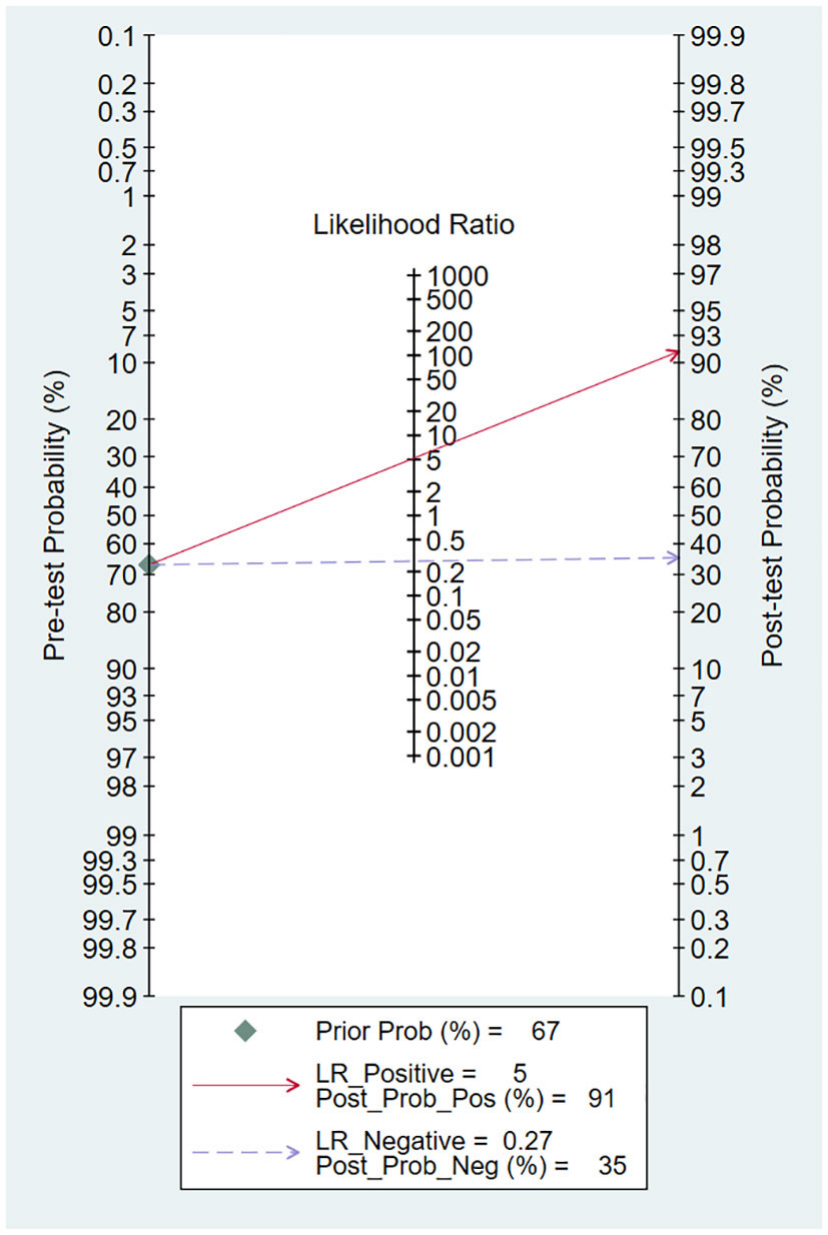
Fagan’s nomogram showing the posttest probability of sepsis.

The HSROC curve for all included studies is depicted in **Figure 4**, and the AUC was 0.88. The pooled sensitivity was 0.77 (95% CI 0.62–0.87), and the pooled specificity was 0.85 (95% CI 0.74–0.92). The pooled PLR was 5.20 (95% CI 2.75–9,84), the pooled NLR was 0.27 (95% CI 0.15–0.48), and the pooled DOR was 19.37 (95% CI 6.71–55.92). Fagan’s nomogram showed that with the pretest probability of 67%, the posttest probability reached 91% and 35% for the positive and negative tests, respectively (**Figure 5**). These results confirmed the high diagnostic efficiency of serum calprotectin in the diagnosis of sepsis. Fagan’s nomogram comprehensively considers PLR and NLR and adjusts the likelihood ratios according to the prior probability of diagnosis for salivary biomarkers. It shows that serum calprotectin could be helpful in diagnosing sepsis.

We performed a heterogeneity analysis of the six studies. The *I^2^* was 71.4% for DOR, indicating that substantial heterogeneity exists among them, while the threshold analysis *p* value was 0.54, giving no evidence of a threshold effect.

We then performed a subgroup analysis to explore the cause of heterogeneity. The subgroup analysis was based on admission category, population setting, and sepsis definition. There are five studies focused on patients treated by physicians and constituted the medical subgroup, while two studies focused on patients undergoing surgery and were termed the surgery subgroup. The pooled DOR was 12.23 (95% CI 3.82–39.15, *I^2^* = 68.5%) and 4.53 (95% CI 1.01–20.38, *I^2^* = 71.2%), respectively. According to the population setting, the six studies were divided into “adult” subgroup and “neonate” subgroup. Pooled DOR was 21.56 (95% CI 6.75–68.83, *I^2^* = 64.5%) and 15.42 (95% CI 1.85–128.6, *I^2^* = 80.5%), respectively. Similarly, two studies used the ACCP/SCCM definition for sepsis and they made up the “ACCP/SCCM” subgroup, while the other four made up the “others” subgroup. Pooled DOR was 39.53 (95% CI 18.19–85.89, *I^2^* = 0.0%) and 12.16 (95% CI 3.37–43.87, *I^2^* = 70.7%), respectively. The results of the subgroup analysis were shown in **Table 2**.

**Table 2:** Results of the subgroup analysis.

Study	Subgroup	n	DOR	95% CI	*I^2^* (%)
Six studies		6	16.08	5.33-48.50	78.3
Admission category	Medical	5	12.23	3.82-39.15	68.5
Population setting	Adult	3	18.18	3.80-86.96	84.2
	Neonate	3	15.42	1.85-128.60	80.5
Sepsis definition	ACCP/SCCM	2	39.53	18.19-85.89	0.0
	Others	4	10.54	2.95-37.00	73.3

DOR, diagnostic odds ratio; ACCP/SCCM, American College of Chest Physicians/Society of Critical Care Medicine.

**Figure 6:**
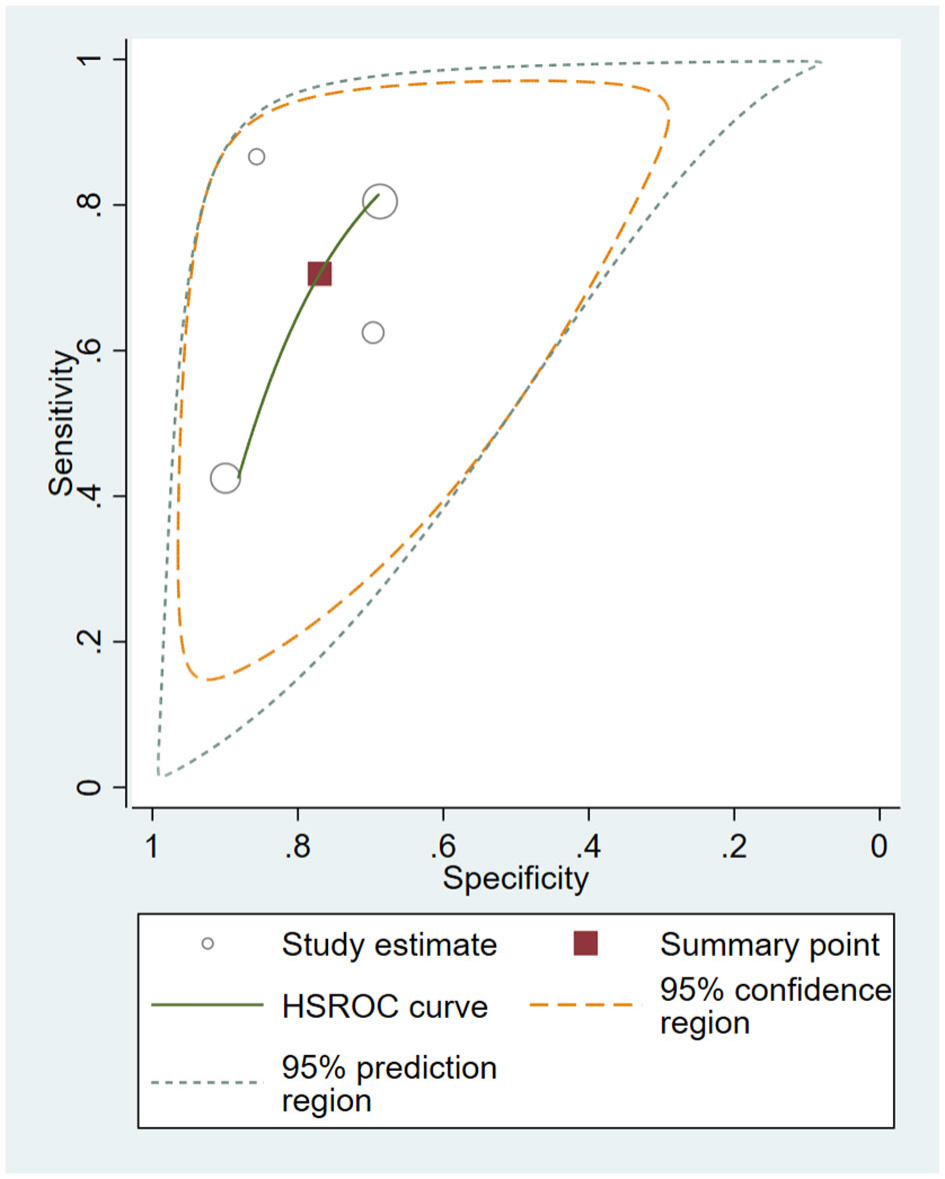
Hierarchical summary receiver operating characteristic (HSROC) curves for four studies.

In the sensitivity analysis, two studies (Canani et al., 2011; Gao et al., 2015) could affect the stability of the pooled results. Omitting these studies, the pooled sensitivity decreased to 0.71 (95% CI 0.51–0.85) and the pooled specificity was 0.77 (95% CI 0.63–0.87). The pooled PLR was 3.07 (95% CI 2.03-4.64), the pooled NLR was 0.38 (95% CI 0.23–0.64), and the pooled DOR was 8.02 (95% CI 4.07–15.80). The HSROC for the four studies was shown in **Figure 6**.

## Discussion

Sepsis is a life-threatening condition caused by a dysregulated host response to infection. Sepsis alters innate and adaptive immune responses and thus hampers immune homeostasis (Delano and Ward, 2016). We now diagnose sepsis with the help of the SOFA score, but methods diagnosing infection are still limited. As a result, there is a great need for early and reliable diagnostic biomarkers for sepsis.

Many studies have suggested that PCT is a biomarker for the early diagnosis of sepsis. However, different pathogens are believed to induce varied levels of PCT, and viral infection or ssystemic inflammatory response syndrome caused by noninfectious diseases can elevate the PCT level in critically ill septic patients (Parli et al., 2018). Several meta-analyses of its diagnostic accuracy in sepsis have been published (Wacker et al., 2013; Ruan et al., 2018; Tan et al., 2019; Li et al., 2021). They reported pooled sensitivities ranging from 0.71 to 0.81, pooled specificities ranging from 0.76 to 0.79, and AUC ranging from 0.80 to 0.87. According to our study, calprotectin is a useful biomarker for its better specificity and similar sensitivity.

Calprotectin is a protein secreted extracellularly from neutrophils and monocytes or is released into pus after cell disruption or death (Rammes et al., 1997; Boussac and Garin, 2000; Voganatsi et al., 2001). The soluble form of calprotectin was found in plasma, urine, body secretions, intestinal fluid, and feces (Pillay et al., 1998). Recently, it has gained great attention for participating in many types of infectious and inflammatory diseases, including inflammatory bowel disease, myocardial infarction, rheumatological diseases, and sepsis (Morrow et al., 2008; Sipponen and Kolho, 2015; Guo et al., 2016; Tydén et al., 2017). Calprotectin regulates immunity and participates in the inflammatory response and antimicrobial activities (Edgeworth et al., 1991; van Zoelen et al., 2009). As a result, calprotectin plays an important role in sepsis-induced alterations in the immune system.

Our results indicated that serum calprotectin on ICU admission could be a practical marker of sepsis. To the best of our knowledge, this is the first meta-analysis reporting the diagnostic value of calprotectin for sepsis on ICU admission, and the results are promising. Although heterogeneity existed, we performed a subgroup analysis and a sensitivity analysis to reduce it.

In the subgroup analysis, calprotectin showed well diagnostic value in both “surgical” and “medical” subgroups, indicating that serum calprotectin could be a potential biomarker to identify sepsis from noninfectious inflammatory disease. Heterogeneity still existed, so admission category was not the main cause of heterogeneity. We then analyzed if the population setting could affect the results, since it was reported that serum calprotectin levels were massively elevated after birth then slowly decreased during the first month of life (Pirr et al., 2017). However, both “neonate” and “adult” subgroups showed a high diagnostic efficacy, suggesting that the population setting was not the main cause of heterogeneity. Research conducted by Gao et al. (2015) and Simm et al. (2016) used the ACCP/SCCM definition for sepsis, and we discovered no heterogeneity in this subgroup. The sepsis definition may be a cause of heterogeneity. Over the past decades, a substantial amount of research improved the recognition and treatment of sepsis, and criteria changed during the process. In 2016, sepsis-3 was developed to further refine it, with an increased focus on recognizing organ dysfunction in the context of infection (Singer et al., 2016). The knowledge on the syndrome is still improving. There is a call for more research to investigate this promising diagnostic marker using a new definition.

Sensitivity analysis showed that studies conducted by Gao et al. (2015) and Canani et al. (2011) could result in heterogeneity, although they demonstrated an excellent diagnostic value of calprotectin in sepsis. Gao et al. (2015) did not report if the sample was enrolled consecutively or randomly, and whether there were patients lost to follow-up was not reported. As for the study by Canani et al. (2011), the sample size of 91 patients was small, and there were 28 patients excluded because of incomplete clinical data. Moreover, they selected neonates with blood culture-proven sepsis and those with the presence of a typical clinical picture of sepsis; this could lead to misclassification. These limitations could cause heterogeneity.

Our meta-analysis has several limitations. First, this meta-analysis included only six studies, and seven other studies associated with this topic were excluded because of insufficient data. Second, significant heterogeneity exists between these studies. As a result, we performed a subgroup analysis and a sensitivity analysis. In the subgroup analysis, sepsis definition turned out to be a source of heterogeneity, but there are no sufficient data to investigate the sepsis-3 definition. The broad and variable definition of sepsis is a common limitation in all sepsis biomarker studies, and the heterogeneities affected the results. Third, all of the studies included selected the test threshold to optimize sensitivity and specificity; it may lead to overestimation of test performance. However, there is no commonly accepted calprotectin threshold for sepsis. This limitation is still inevitable.

## Conclusions

In conclusion, serum calprotectin on ICU admission plays an important role in the timely diagnosis of sepsis. However, further studies are required to confirm this finding.

## Data availability statement

The original contributions presented in the study are included in the article. Further inquiries can be directed to the corresponding author.

## Author contributions

R-YG contributed to study design, search strategy, screening of studies, data extraction, and statistical analysis, as well as writing the manuscript. H-MJ contributed to screening of studies and data extraction, as well as assisted in editing the manuscript. Y-ZH contributed to search strategy, screening of studies, and data extraction. B-SQ performed the quality assessment and PY synthesized the data. XKZ and WXL contributed in revising the manuscript. L-FH conceived of the study, contributed to study design and provided general supervision. All authors contributed to the article and approved the submitted version.

## Conflict of interest

The authors declare that the research was conducted in the absence of any commercial or financial relationships that could be construed as a potential conflict of interest.

## Publisher’s note

All claims expressed in this article are solely those of the authors and do not necessarily represent those of their affiliated organizations, or those of the publisher, the editors and the reviewers. Any product that may be evaluated in this article, or claim that may be made by its manufacturer, is not guaranteed or endorsed by the publisher.
